# Establishment of fast-growing serum-free immortalised cells from Chinese hamster lung tissues for biopharmaceutical production

**DOI:** 10.1038/s41598-020-74735-0

**Published:** 2020-10-19

**Authors:** Noriko Yamano-Adachi, Rintaro Arishima, Sukwattananipaat Puriwat, Takeshi Omasa

**Affiliations:** 1 Graduate School of Engineering, Osaka University, 2-1, Yamadaoka, Suita, Osaka 565-0871 Japan; 2 Manufacturing Technology Association of Biologics, 7-1-49, Minatojima-Minamimachi, Chuo-ku, Kobe, Hyogo 650-0047 Japan

**Keywords:** Biotechnology, Cell biology

## Abstract

Chinese hamster (*Cricetulus griseus*) ovary-derived Chinese hamster ovary (CHO) cells are the most commonly used mammalian hosts for the industrial production of recombinant therapeutics because of their ability to fold, assemble, and perform post-translational modifications, such as glycosylation, on proteins. They are also valuable for their ability to grow in serum-free suspension cultures. In this study, we established a cell line derived from lung tissue of Chinese hamsters, named Chinese hamster lung (CHL)-YN cells. The biosafety of CHL-YN cells was confirmed by in vitro sterility testing, mycoplasma detection, and reverse transcriptase assays. One of the key characteristics of CHL-YN cells was their doubling time of 8.1 h in chemically defined culture medium; thus, they proliferate much faster than conventional CHO cells and general mammalian cells. Transgenes could be introduced into CHL-YN cells with high efficiency. Finally, between 50% to > 100% of the amount of glycosylated immunoglobulin G (IgG)1 produced by CHO-K1 cells was produced by CHL-YN cells over a shorter period of time. In summary, fast-growing CHL-YN cells are a unique cell line for producing recombinant proteins.

## Introduction

Chinese hamster ovary (CHO) cells are an epithelial-like cell line established by Dr. Puck in 1957^1^ . CHO cells are widely used in the biopharmaceutical industry to produce recombinant proteins such as immunoglobulin G (IgG)^2^. One of the characteristics of CHO cells is that they can adapt to serum-free medium and can be cultured in chemically defined medium without animal-derived components^3–5^. When CHO cells are cultured in serum-free medium, the cells detach from the surface and float, which enables large-scale high density cell cultures in bioreactors. Additionally, CHO cells have advantages over other hosts, such as *Escherichia coli* and *Saccharomyces cerevisiae*, in terms of the correct folding, assembly, and post-translational modifications such as glycosylation of proteins^6,7^ . Previous knowledge about the safety of viruses in CHO cells is also an advantage of using them in these settings. For example, 44 human pathogenic viruses, including HIV, influenza, polio, herpes, and measles, have been shown to not replicate in CHO cells^8,9^. Additionally, the risk of viral infection is lower than when using human-derived cells. Conversely, they have the disadvantage of growing more slowly than non-mammalian host cells^10,11^. Recently, biopharmaceuticals have been increasing their market share, and the demand for CHO cells has also increased^2,12^.

CHO cells are not the only cell line that has been derived from Chinese hamsters. It has been reported that cells derived from Chinese hamster lungs proliferate well in vitro^13,14^. It has also been reported that there is a total absence of detectable cell cycle Gap 1 (G1) phase in cultured Chinese hamster lung cells^15^. This is consistent with studies that showed some pulmonary-derived bovine fibroblasts are more proliferative in hypoxic environments, although the response of cells to hypoxia varied from clone to clone^16^.

In this study, we established a new cell line from primary cultures of Chinese hamster lung tissue to develop a highly productive host cell that can be a faster growing alternative to conventional CHO cells. To the best of our knowledge, this is the first time a Chinese hamster lung cell line has been established with a clear history of cell culture from seeding tissue to becoming adapted to grow in chemically defined medium; thus, these cells could become important host cells for the biopharmaceutical industry.

## Results

### Establishment of a serum-free, immortalised cell line from Chinese hamster lung tissue

**Figure 1:**
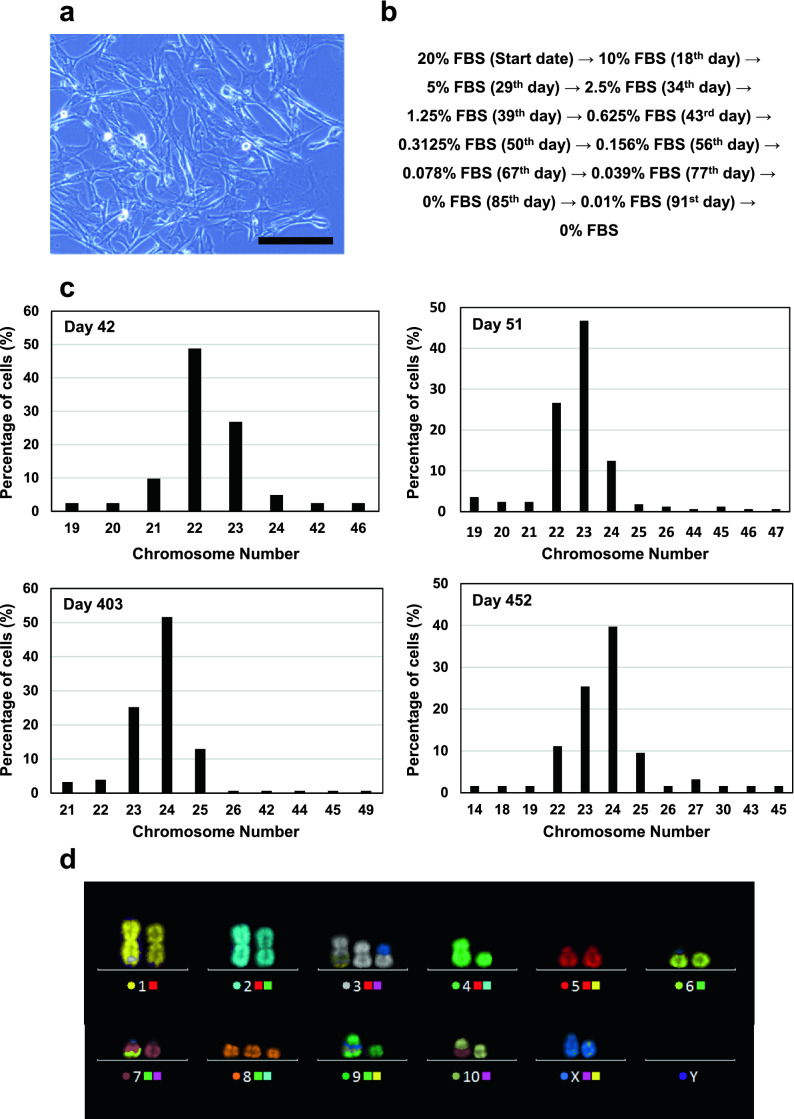
Acquisition of serum-free immortalised cells from Chinese hamster lung tissue. **(a)** Fibroblast-like morphologies of CHL-YN cells on day 10 of culture. Primary cultured cells in IMDM medium containing 20% FBS were imaged under an Olympus CKX 41 microscope using a Wraycam colour CMOS camera NF 300; scale bar: 200 μm. **(b)** Detailed procedure of the serum-free adaptation of CHL-YN cells. **(c)** Distribution of the number of chromosomes in CHL-YN cells at 42, 51, 403, and 452 days of culture. Chromosome numbers were counted for 41 cells (day 42), 169 cells (day 51), 155 cells (day 403), and 63 cells (day 452). (d) Karyotype of CHL-YN cells on day 452 shown in pseudo colour image. The colour of the circle to the left of the number indicates the pseudo colour of each chromosome. The colour of the square to the right of the number indicates the combination of fluorochromes of the probes used to recognise each chromosome.

The primary cultured cells obtained from Chinese hamster lung tissue primarily showed a fibroblast-like morphology in Iscove’s modified Dulbecco’s medium (IMDM) containing foetal bovine serum (FBS) (Fig. 1a). Expanded cells were cultured with gradually decreasing serum concentrations to adapt the cells for chemically defined medium. Details of the serum-free adaptation procedure are described in Fig. 1b. Cells cultured in chemically defined medium at 360 and 367 days of culture were subjected to an in vitro assay to detect viral contamination, sterility testing by direct inoculation, and a mycoplasma detection test. Cells that passed these tests (at culture date of 360) were deposited to Riken BioResource Research Center (RIKEN BRC, Tsukuba, Japan; number RCB5004), and we named the cells “Chinese hamster lung (CHL)-YN”. Additionally, a reverse transcriptase activity assay was performed on day 402 cells to confirm negative results. The CHL-YN cells were naturally transformed and immortalised. The percentage of aneuploid cells increased during the immortalisation process (Fig. 1c). The karyotype of cells on culture day 452 was different from the original Chinese hamster karyotype (Fig. 1d). Figure 1d shows the karyotype most commonly observed at day 452; in this cell, there is an extra chromosome 8 along with translocations between chromosomes 1 and 3, 3 and X, 7 and 6, 9 and 3, and 10 and 7. Interestingly, some translocations with the same pattern as that seen in the CHO-DG44- and CHO-K1-derived cells were observed in cells on culture day 452 ^17^ .

### Comparison of CHO-K1 and CHL-YN (with and without FBS) cells

**Figure 2:**
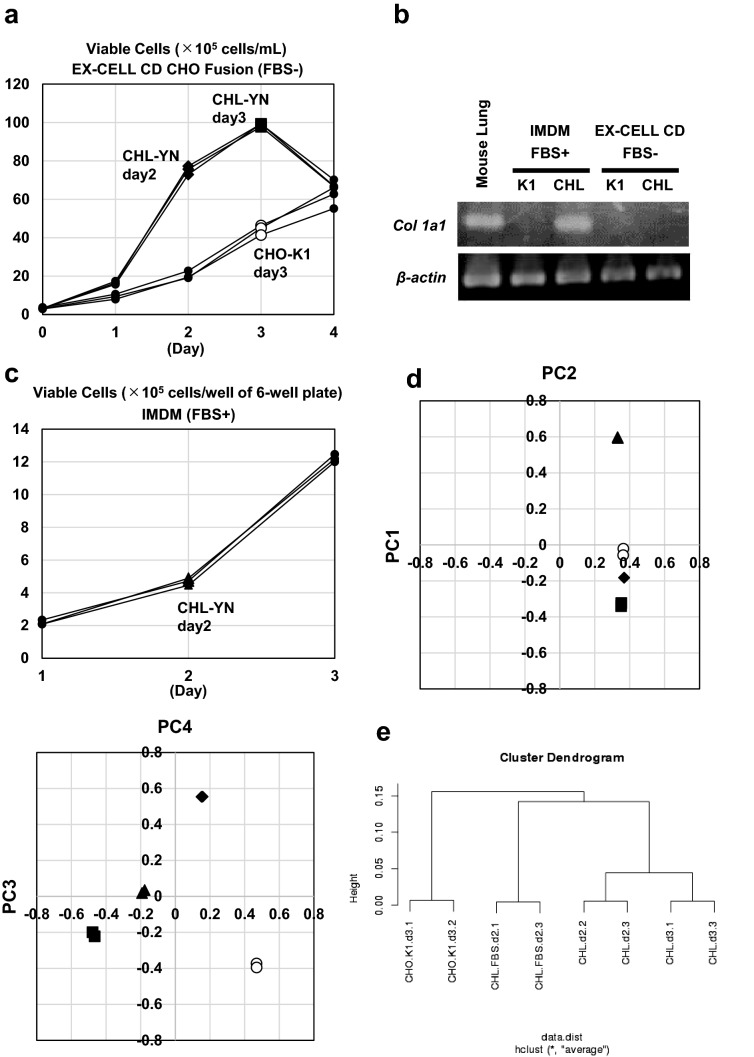
Comparison of CHO-K1 and CHL-YN cells with and without FBS. **(a)** Viable densities of cells cultured in EX-CELL CD CHO Fusion medium containing 6 mM l-glutamine (FBS −). Cell samples were taken at the indicated time points: a white circle represents “CHO-K1 day 3”; a black diamond represents “CHL-YN day 2”; and a black square represents “CHL-YN day 3”. **(b)** *Col1a1* mRNA levels in CHO-K1 and CHL-YN cells. First strand cDNA from murine lung tissue was used as the control. **(c)** Number of viable cells cultured in a well of a 6-well plate in IMDM medium (FBS +). Cell samples for “CHL-YN day 2” were taken at the point indicated by a black triangle. **(d)** Results of principal component analysis. White circles indicate the results of “CHO-K1 (EX-CELL CD CHO Fusion, FBS −) day 3”, black diamonds indicate the results of “CHL-YN (EX-CELL CD CHO Fusion, FBS −) day 2”, black squares indicate the results of “CHL-YN (EX-CELL CD CHO Fusion, FBS −) day 3”, and black triangles indicate the results of “CHL-YN (IMDM, FBS +) day 2”. **(e)** Clustering analysis. Clustering was performed using the group mean method after defining the distance between samples by the Spearman rank correlation coefficient.

The growth of CHL-YN cells in chemically defined medium was significantly faster than that of CHO-K1 cells (Fig. 2a). The doubling time of CHL-YN was 8.1 h (specific growth rate: 0.086 h^−1^) in EX-CELL CD CHO Fusion medium containing 6 mM L-glutamine (Fig. 2a). The cell cycle analysis showed that CHL-YN cells has a lower percentage of G0/G1 cells than CHO-K1 cells (Table 1). *Col1a1*, which is highly expressed in fibroblasts, was found to be expressed when cells were cultured in serum-containing medium (Fig. 2b, Supplementary Fig. 1). Conversely, *Col1a1* was undetectable in cells cultured in serum-free chemically defined medium after the serum-free adaptation (Fig. 2b, Supplementary Fig. 1). To compare the characteristics of CHO-K1 and CHL-YN cells (with and without FBS), the two cell lines were subjected to RNA-seq analysis. Cell samples were collected at the time points indicated in Fig. 2a,c: on day 3 for CHO-K1 cells (EX-CELL CD CHO Fusion, FBS −), on days 2 and 3 for CHL-YN cells (EX-CELL CD CHO Fusion, FBS −), and on day 2 for CHL-YN cells (IMDM, FBS +). From principal component analysis (Fig. 2d) and clustering analysis (Fig. 2e), the different media (with or without FBS) had a greater difference than that of the cell growth phase in CHL-YN cells. Additionally, CHO-K1 cells showed different characteristics compared with CHL-YN cells (Fig. 2d,e). Spearman's correlation analysis showed that the mean distant matrix from the CHL-YN cells on day 2 (EX-CELL CD CHO Fusion, FBS −) were 0.96 for CHL-YN cells on day 3 (EX-CELL CD CHO Fusion, FBS −), 0.90 for CHL-YN cells on day 2 (IMDM, FBS +), and 0.88 for CHO-K1 cells on day 3 (EX-CELL CD CHO Fusion, FBS −) (Fig. 2e).

**Table 1:** Cell cycle analysis.

		Percentage	Cell count				
			Field 1	Field 2	Field 3	Field 4	Total
CHO-K1	G0/G1	72%	279	297	347	364	1287
	S/G2/M	28%	88	128	127	147	490
CHL-YN	G0/G1	49%	150	160	172	206	688
	S/G2/M	51%	160	203	162	183	708

The assay used a redox dye that is imported by live cells. Following dye uptake and incubation, a distinct colour change occurs within cells, with particular colour changes being associated with cells in the G1 (Gap 1), S (Synthesis), G2 (Gap 2), and M (Mitosis) phases of the cycle.

**Figure 3:**
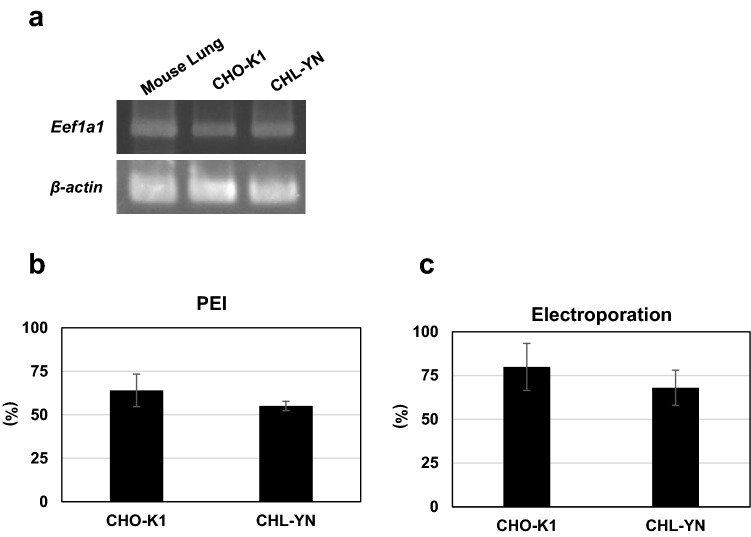
Constructing transgene expressing cells. **(a)** *Eef1a1* mRNA expression in CHO-K1 and CHL-YN cells. mRNA was extracted from cells cultured in EX-CELL CD CHO Fusion medium containing 6 mM l-glutamine. First strand cDNA from murine lung tissue was used as the control. **(b)** Transfection efficiencies using PEI. The positive rate of green fluorescent protein (GFP) expression was measured by flow cytometry. Values are expressed as mean ± standard deviation (n = 3) **(c)** Transfection efficiencies using electroporation. The positive rate of GFP expression was measured by microscopic observation. Values are expressed as mean ± standard deviation (n = 4).

Table 2 shows the top 10 most highly expressed genes in each cell group after reads per kilobase of exon per million mapped reads (RPKM) normalisation. The expression of eukaryotic translation elongation factor 1 alpha 1 (*Eef1a1*), the promoter of which is commonly used in transgene expression, was the highest in CHO-K1 and CHL-YN suspension cultures (EX-CELL CD CHO Fusion, FBS −), and the second highest in CHL-YN adherent culture (IMDM, FBS +) (Table 2). *Eef1a1* expression in EX-CELL CD CHO Fusion medium was also confirmed by RT-PCR (Fig. 3a, Supplementary Fig. 2).

**Table 2:** Gene expression rankings of each cell line after RPKM (reads per kilobase of exon per million mapped reads) normalisation.

	CHO-K1 EX-CELL CD FBS − day3	CHL-YN EX-CELL CD FBS − day2	CHL-YN EX-CELL CD FBS − day3	CHL-YN IMDM FBS + day2
1	eEF1a1 3504.18	eEF1a1 3549.87	eEF1a1 7070.71	Actin, beta 3191.55
2	Actin, beta 2553.90	RPS2 3166.78	RPS2 4134.01	eEF1a1 2506.23
3	RPL23A (X3) 2340.60	MT2 2711.28	FTH1 3797.56	ACTG1 (X4) 1666.11
4	RPL23A (X4) 2056.29	GAPDH 2023.31	RPS18 (X2) 3098.57	RPS2 1576.86
5	GAPDH 1838.53	NPM (X3) 1760.33	RPLP0 (X1) 2913.81	FTH1 1543.47

Numbers shown in the table are the RPKM values of each gene.

### Transfection efficiency

Transfection efficiency is important when using cells as hosts for recombinant protein production. As a result of our examination, a high transfection efficiency, the same as for CHO-K1 cells, was found for CHL-YN cells by the following methods: (1) polyethylenimine (PEI)-based transfection, for which the efficiency of CHL-YN cells exceeded 50% (Fig. 3b); and (2) electroporation, which achieved an efficiency of approximately 70% (Fig. 3c). These protocols are described in detail in the “Methods”. Although the percentage of cells in which exogenous genes were successfully inserted into the chromosome is unknown, the survival rate of cells that underwent drug resistance selection with 5 µg/mL puromycin was similar between CHL-YN and CHO-K1 cells.

### Production of humanised IgG1 using CHL-YN cells as hosts

**Figure 4:**
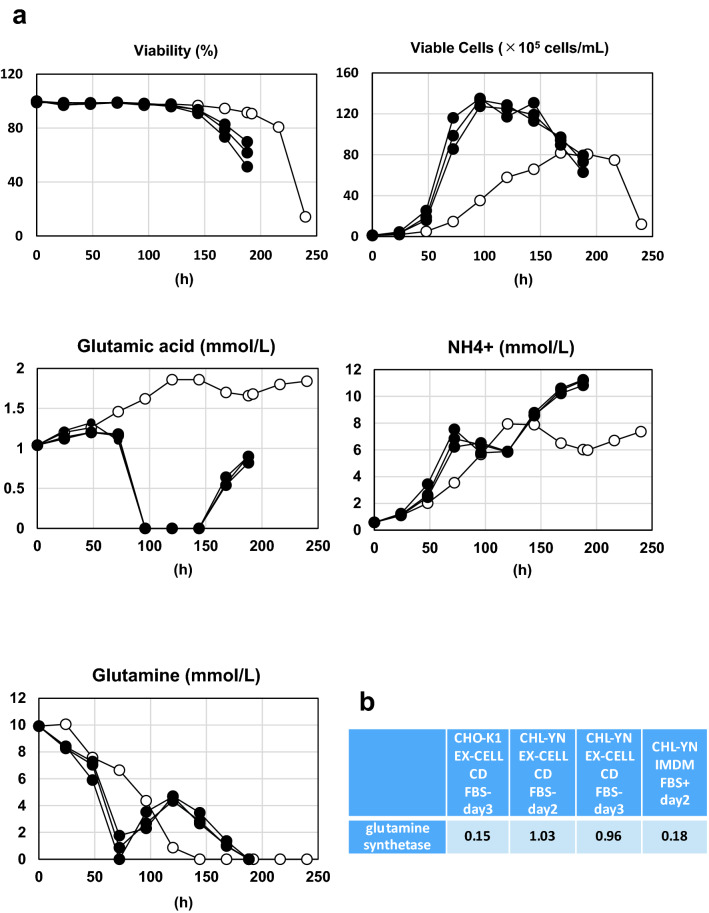
Metabolite analysis in CHL-YN cells. **(a)** Cells were seeded at a density of 1.2 × 10^5^ cells/mL in 500-mL Erlenmeyer flasks (100-mL culture). Cells were cultured in EX-CELL CD CHO Fusion medium containing 8 mM l-glutamine. Concentrations of glutamic acid, ammonium ion, and glutamine were determined in cell culture supernatants, in addition to viability and viable cell density during batch cultures. White circles indicate CHO-K1 cells, and black circles indicate CHL-YN cells. **(b)** RPKM normalised RNA-seq data of glutamine synthetase.

**Figure 5:**
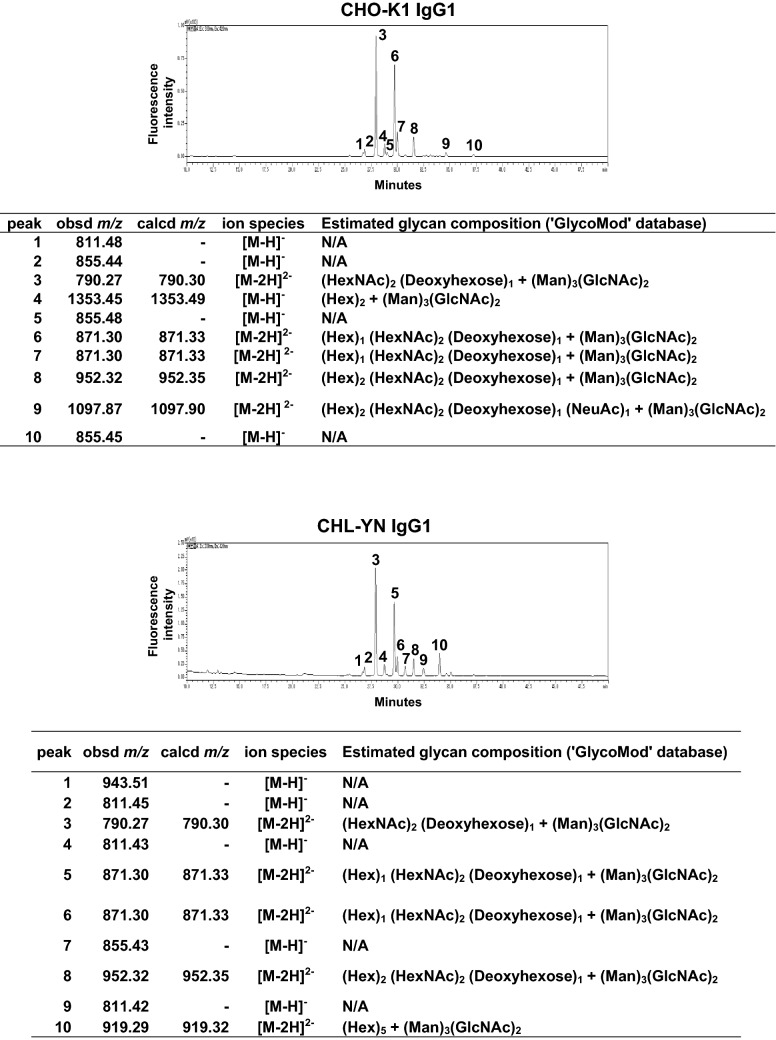
Top 10 peaks of estimated N-glycan modification to IgG1. The N-glycan profiles of IgG1 purified from cell culture supernatants on day 9 of batch culture from IgG1-expressing CHO-K1 cells and on day 7 of batch culture from IgG1-expressing CHL-YN cells were analysed by HPLC–MS to determine the glycan structures. Glycan compositions were estimated by the GlycoMod tool, and those recorded in the UniCarbKB database were extracted.

Recombinant IgG1-producing CHL-YN and CHO-K1 cells were constructed using PEI-based transfection. Stably producing cell pools resulting from drug resistance selection with 5 µg/mL puromycin were used for subsequent analysis. The specific growth rates of CHL-YN and CHO-K1 cells tended to decrease slightly when IgG1 was expressed (Fig. 4a, Table 3). In addition to viability and viable cell density, concentrations of glutamic acid, ammonium ion, and glutamine were determined in cell culture supernatants every 24 h after seeding the cells in batch cultures. Interestingly, the amount of glutamine increased after it was consumed, and the amount of glutamic acid decreased at approximately the same time (Fig. 4a). RNA-seq data showed that the expression of glutamine synthetase, an enzyme that uses ATP to catalyse the condensation of glutamate with ammonia to form glutamine, was higher in CHL-YN cells (EX-CELL CD CHO Fusion, FBS −) compared with CHO-K1 cells (EX-CELL CD CHO Fusion, FBS −) and CHL-YN cells (IMDM, FBS +) (Fig. 4b). Recombinant IgG1 expression was detected when CHL-YN was used as a host. One of the results of the IgG production test in batch culture is shown in Table 3. Between approximately 50% to > 100% of the amount of IgG1 that was produced by CHO-K1 cells during batch culture was achieved by CHL-YN cells in a shorter period of time. Liquid chromatography-mass spectrometry (LC–MS)-based N-glycan profiles of IgG1 showed the same high peaks between CHO-K1 and CHL-YN products (Fig. 5).

**Table 3:** IgG1 productivity assessment in 500 mL Erlenmeyer flask batch cultures.

Cell type	CHL-YN	CHO-K1
Specific growth rate (h^−1^)	0.0645 ± 0.0012	0.0326 ± 0.0005
Doubling time (h)	10.74 ± 0.20	21.29 ± 0.34
Specific production rate (pg cell^−1^ day^−1^)	0.2554 ± 0.0115	0.1239 ± 0.0232
Final IgG1 concentration (mg/L)	7.13 ± 0.29	6.05 ± 0.73

Cells were seeded at a density of 3 × 10^5^ cells/mL in 500-mL Erlenmeyer flasks (100-mL culture). Cells were cultured in EX-CELL CD CHO Fusion medium containing 6 mM l-glutamine. To calculate specific growth rates and doubling times, the number of CHL-YN cells was counted 0, 5, 8, 16, 24, 33, 40, 48, and 57 h after the initial seeding, and the number of CHO-K1 cells was counted 0, 24, 48, 72, and 96 h after the initial seeding. To calculate specific production rates, cultured medium was taken every 24 h after the initial seeding.

## Discussion

In this study, we report the establishment of rapidly growing CHL-YN cells from Chinese hamster lung tissue as a candidate new host cell for producing recombinant proteins. The N-linked glycosylation of biopharmaceutical human IgG affects antibody-dependent cell-mediated cytotoxicity (ADCC) and complement-dependent cytotoxicity (CDC) activities, which are the main mechanisms of action of antibody drugs^18–20^. N-glycan profiles of IgG1 produced from CHL-YN cells showed the same high peak as that from CHO-K1 cells, indicating similar glycosylation patterns to that of IgG1 produced in CHO cells. From our results, the introduction of transgenes is possible at high efficiency in CHL-YN cells, and the EF1-α promoter, a common promoter for high expression in CHO cells, can also be used in CHL-YN cells. Additionally, the biological safety of CHL-YN cells, although to minimal levels, was investigated and found to be applicable for industrial host cells.

CHL-YN cells differ from CHO cells in tissue of origin and in primary cell morphology. Cells are known to change their properties in response to their environment^21^ , and our results also showed that culture medium conditions dramatically changed the gene expression profiles of CHL-YN cells, including that some of the fibroblast characteristics that were originally found in CHL-YN cells were lost after culture in serum-free medium. Using the same chemically defined medium for CHO-K1 and CHL-YN cells, the overall correlation difference was greater for CHO-K1 and CHL-YN cells than for differences between the culture conditions of CHL-YN cells. For CHL-YN cells cultured in EX-CELL CD CHO Fusion medium containing 6 mM l-glutamine, our analysis of the culture supernatant constituents revealed that, after the added glutamine was consumed, the concentration of glutamine increased, and glutamic acid was consumed. Additionally, the results of a comprehensive gene expression analysis further suggest that CHL-YN cells gained the expression of glutamine synthase under serum-free conditions. We have been culturing CHL-YN cells for over four years since the occurrence of this serum-free adaptation, and the phenomenon has been maintained. It is unknown how CHL-YN cells acquired the expression of glutamine synthase following serum-free culture; however, another study similarly reported that the metabolisms of glutamine and glutamate were found to be differentially regulated after adaptation to suspension growth in CHO cells^22^. Notably, glutamic acid can be used instead of glutamine when culturing CHL-YN cells, which is advantageous because medium preparation is easier when heat-labile glutamine is not required. It is necessary and interesting to perform assays that highlight the differences between CHL-YN and CHO cells. Additionally, through analysing CHL-YN cells, it is possible to develop studies for CHO-K1 cells. For example, by comparing the newly constructed CHL-YN with CHO-K1 cells, which have been continuously cultivated in vitro for decades, it is possible to distinguish stable/unstable chromosomes and to elucidate the cause of chromosomal instability, a characteristic of Chinese hamster-derived cells.

CHL-YN cells were characterised by rapid growth compared with general mammalian cells^1,14,23^ . The rate of cell growth is an important factor that shortens the duration of large-scale industrial cell culture. To take advantage of this, it is ideal if cells can be controlled not only to grow rapidly, but also to not overgrow after a certain cell density has been reached. For example, the temperature shift strategy is a famous way to suppress cell proliferation in CHO and CHL cell cultures^14,24^. Additionally, we have previously found that epigallocatechin-3-gallate (EGCG), which induces G0/G1 cell cycle arrest, improves recombinant IgG1 productivity in CHO cells^25^. This strategy could be applied to control the proliferation of CHL-YN cells, although conditions such as EGCG concentration may differ. Furthermore, higher specific production rates may be obtained by controlling DO and pH using a bioreactor or by performing fed-batch or perfusion culture. By optimising cell culture conditions, we expect that CHL-YN cells will be widely used as host cells for recombinant proteins in the future.

## Methods

### Statements

All methods were carried out in accordance with relevant guidelines and regulations. All experimental protocols were approved by Osaka University and Manufacturing Technology Association of Biologics**.**

### Primary cell culture

Primary cultures were performed by disseminating the lung tissue of female Chinese hamsters (Charles River Laboratories Japan, Inc., Kanagawa, Japan) that had been cut into 1-mm squares into IMDM (Sigma-Aldrich, St. Louis, MO, USA) containing 20% FBS (#172012, lot 12E183, Sigma-Aldrich). Cells were cultured in 100-mm dishes at 37 °C with 5% CO_2_. Proliferating cells were maintained by standard passaging.

### Cell culture and adaptation to serum-free medium

Once expanded, the cells were cultured with gradually decreasing serum concentrations in six-well plates. Passages were continued with custom-made serum-free medium, by replacing half of the serum-containing IMDM medium. The custom-made serum-free medium consisted of Top2 (Irvine Science, Santa Ana, CA, USA), HyClone CDM4CHO (GE Healthcare, Chicago, IL, USA), and EX-CELL CD Hydrolysate Fusion (Sigma-Aldrich) media, supplemented with 6 mM l-glutamine (Wako Pure Chemical Industries, Ltd., Osaka, Japan). Finally, the cells were acclimated to chemical differential medium, EX-CELL CD CHO Fusion (#14365C, Sigma-Aldrich) medium containing 6 mM l-glutamine (Wako Pure Chemical Industries, Ltd.). After adaptation, the cells were cultured at 37 °C in 125-mL Erlenmeyer flasks (Corning Inc., Corning, NY, USA) with shaking at 80 rpm, 5% CO_2_, and 80% humidity in an orbital Climo Shaker ISF1-X (Kuhner Shaker, Inc., Basel, Switzerland) as described in a previous report^25^. An automated cell analyser (Vi-cell XR, Beckman Coulter, Inc., Brea, CA, USA) was used to analyse total/viable cell concentrations^25^. CHO-K1 (ATCC CCL-61) cells (American Type Culture Collection, Manassas, VA, USA) adapted to EX-CELL CD CHO Fusion medium in the same manner as CHL-YN cells were used for comparison.

### Biosafety testing

An in vitro assay to detect viral contamination, sterility testing by direct inoculation, and a mycoplasma detection test were performed at BioReliance Ltd. (Rockville, MD, USA). Detection of reverse transcriptase by the real-time fluorescent product-enhanced reverse transcriptase (F-PERT) assay was performed at SGS Vitrology Ltd. (Aberdeen, Scotland).

### Chromosome count and karyotype analyses

Cells in exponential growth phase were used to prepare metaphase chromosome spreads as described previously^26^. Chromosome spreads on glass slides were stained with 4′, 6-diamidino-2-phenylindole dihydrochloride (DAPI) to count chromosome numbers. Karyotypes were determined using the multicolour in situ hybridisation (mFISH) method. This method uses 12 painting probes, 12XCHamster (Metasystems, Altlussheim, Germany) specific for the 12 different Chinese hamster chromosomes, each labelled with different fluorochromes. Chromosome images were taken under an Axio Imager.Z2 fluorescence microscope (Carl Zeiss, Oberkochen, Germany). Karyotypes were analysed using the Isis fluorescence imaging system (Metasystems).

### Cell cycle analysis

Cells were cultured in EX-CELL CD CHO Fusion medium containing 6 mM l-glutamine. Cell cycle analysis was performed on cells growing in growth phase (passaged to fresh medium on the previous day) with the Cell-Clock Cell Cycle Assay Kit (Biocolor Ltd., County Antrim, UK). Cell numbers were counted according to colour by microscopic observation (BZ-X700, Keyence Corporation, Osaka, Japan).

### RT-PCR analysis

Total RNA was extracted from cultured cells using the High Pure RNA Isolation Kit (Roche, Ltd., Basel, Switzerland), and then 1 μg of total RNA was used for cDNA synthesis. Reverse transcription was performed using the PrimeScript First Strand cDNA Synthesis kit (Takara Bio Inc., Shiga, Japan). First strand cDNA from murine lung cells was purchased from GenoStaff Co., Ltd. (Tokyo, Japan). The purchased first strand cDNA was diluted two-fold, and samples synthesised using the PrimeScript First Strand cDNA Synthesis kit were diluted 30-fold diluted, of which 1 μL of each was used as a template. PCR amplification was carried out with KOD-Plus-Neo (Toyobo Co., Ltd., Osaka, Japan) for *Col1a1* and 2 × PrimeSTAR HS (Premix) (Toyobo Co., Ltd.) for *Eef1a1*. The gene-specific primers used for PCR amplification were following: *Col1a1*, 5′-TGCCAAAGGAGATGCTGGTC-3′, and 5′-ACCAGCAATACCAGGAGCAC-3′; *Eef1a1*, 5′-ATTGATGCCCCAGGACACAGAGAC-3′, and 5′-GGTTCAGGATAATCACCTGAGCAG-3′; and *β-actin* (internal control), 5′-ACCTCATGAAGATCCTGACC-3′, and 5′-CAATGCCTGGGTACATGGTG-3′. The primers were designed with homologous sequences in *Mus musculus* and *Cricetulus griseus*.

### RNA-seq analysis

Total RNA was extracted from cells using the RNeasy Mini Kit (Qiagen, Hilden, Germany). Extracted total RNA was sent to Bioengineering Lab. Co., Ltd. (Kanagawa, Japan) for RNA-seq analysis. Principal component analysis was performed using R's factorial with reads per kilobase of exon per million mapped reads (RPKM)-normalised data. Clustering analysis was performed using R's hclust with count data. After defining the distance between samples with Spearman's rank correlation coefficient, clustering was performed using the group mean method.

### Transfection

Cells were cultured in EX-CELL CD CHO Fusion medium containing 6 mM l-glutamine. Expression vectors were transfected using Polyethylenimine Max (Mw 40,000)-High Potency Linear PEI (Cosmo Bio Co., Ltd., Tokyo, Japan) or the Neon Transfection System (Thermo Fisher Scientific, Inc., Waltham, MA, USA). Transfection with PEI was performed in the following protocol. Cells were passaged 2 days before transfection at 5 × 10^4^ cells/mL for CHL-YN, or 5 × 10^5^ cells/mL for CHO-K1. The day of transfection, the cells were prepared at 1 × 10^6^ cells in 1 mL of warmed HyClone Hycell Trans FX-C media (GE Healthcare) containing 6 mM L-Glutamine per well of the 6-well plate. Then, 2.5 μg of DNA and 7.5 μL of PEI were used per well of the 6-well plate, with the total amount of DNA, PEI, and Opti-Pro SFM (Thermo Fisher Scientific, Inc.) being 50 μL per well. DNA was mixed with Opti-Pro SFM and incubated on ice for 5 min. Then, PEI was added and allowed to react at room temperature for 10 min with occasional turning, after which, the mixture was added to each well. Transfection with the Neon Transfection System was performed according to the instructions at 1750 V, Width: 10, Pulses: 3. For the Neon system, 1 μg of DNA was used for 5 × 10^5^ cells per well of a 6-well plate. Transfection efficiencies were measured after enhanced green fluorescent protein (*eGFP*) expression vectors^27^ were introduced by flow cytometry (BD FACSVerse, Becton, Dickinson Co., Franklin Lakes, NJ, USA) or microscopic observation (BZ-X700, Keyence Corporation).

### Construction of recombinant humanised IgG1-producing cells

Heavy- and light-chain genes were ligated into the Mammalian PowerExpress System (Toyobo Co., Ltd.), and this construct was used as the antibody expression vector^28^. Endotoxins of the plasmid were removed using MiraCLEAN Endotoxin Removal Kit (Mirus Bio LLC, Madison, WI, USA) twice after it was linearised with *SspI*, and then the plasmids were transfected into CHL-YN and CHO-K1 cells using PEI as described above. IgG-expressing cells were selected and cultured in medium containing 5 μg/mL puromycin (InvivoGen, San Diego, CA, USA).

### Kinetic parameters

The specific growth and production rates were calculated as follows^29^. Glucose, lactate, glutamine, glutamic acid, and ammonium ion concentrations in the cell supernatants were determined by BioProfile 400 (Nova Biomedical Corp., Waltham, MA, USA). Recombinant protein concentrations in the cell supernatants were determined by a sandwich enzyme-linked immunosorbent assay (ELISA), as described in a previous report^30^. Briefly, a goat anti-human IgG-Fc fragment (Bethyl Laboratories, Montgomery, TX, USA) was used as the capture antibody, and a horseradish peroxidase (HRP)-conjugated goat anti-human IgG-Fc fragment (Bethyl Laboratories) was used as the detection antibody. Antibody concentrations were calculated by a standard curve that was made using purified monomeric humanised IgG1 produced from CHO cells.

### N-Glycan analysis of IgG1

It was performed as described in a previous report ^25^. IgG1 purification from the cell culture supernatants and labelling of released N-glycans with 2-aminobenzamide were performed by EZGlyco (Sumitomo Bakelite Co. Ltd., Tokyo, Japan). Prepared samples were analysed by high-performance liquid chromatography (HPLC) and mass spectrometry (MS) at Sumitomo Bakelite Co. Ltd.

## Supplementary information

Supplementary Information.

## References

[1] Puck, T. T., Cieciura, S. J., Robinson, A. Genetics of somatic mammalian cells. III. Long-term cultivation of euploid cells from human and animal subjects. *J. Exp. Med.* **108,** 945–956 (1958). 10.1084/jem.108.6.945 PMC2136918 13598821

[2] De Jesus M, Wurm FM (2011). Manufacturing recombinant proteins in kg-ton quantities using animal cells in bioreactors. Eur. J. Pharm. Biopharm..

[3] Wurm FM (2013). CHO quasispecies—Implications for manufacturing processes. Processes..

[4] Ham, R. G. Clonal growth of mammalian cells in a chemically defined, synthetic medium. *Proc. Natl. Acad. Sci. U.S.A.* **53,** 288 (1965). 10.1073/pnas.53.2.288 PMC219509 14294058

[5] Traustason B, Cheeks M, Dikicioglu D (2019). Computer-aided strategies for determining the amino acid composition of medium for Chinese hamster ovary cell-based biomanufacturing platforms. Int. J. Mol. Sci..

[6] Omasa T, Onitsuka M, Kim WD (2010). Cell engineering and cultivation of Chinese hamster ovary (CHO) cells. Curr. Pharm. Biotechnol..

[7] Thak, E. J., Yoo, S. J., Moon, H. Y., Kang, H. A. Yeast synthetic biology for designed cell factories producing secretory recombinant proteins. *FEMS Yeast Res.* **20,** foaa009 (2020). 10.1093/femsyr/foaa009 32009173

[8] Wiebe, M., Becker, F., Lazar, R., May, L., Casto, B., Semense, M., *et al*. A multifaceted approach to assure that recombinant tPA is free of adventitious virus. in *Advances in Animal Cell Biology and Technology for Bioprocesses.* (ed. Spier, R. E.) 68–71 (Butterworths, 1989).

[9] Jayapal KP, Wlaschin KF, Hu W, Yap MG (2007). Recombinant protein therapeutics from CHO cells-20 years and counting. Chem. Eng. Prog..

[10] Rosano GL, Ceccarelli EA (2014). Recombinant protein expression in *Escherichia coli*: Advances and challenges. Front. Microbiol..

[11] Bhattacharya S, Esquivel BD, White TC (2018). Overexpression or deletion of ergosterol biosynthesis genes alters doubling time, response to stress agents, and drug susceptibility in *Saccharomyces cerevisiae*. MBio..

[12] Kaplon H, Muralidharan M, Schneider Z, Reichert JM (2020). Antibodies to watch in 2020. MAbs..

[13] Kao, F. T., Puck, T. T. Genetics of somatic mammalian cells. IV. Properties of Chinese hamster cell mutants with respect to the requirement for proline. *Genetics.* **55,** 513–524 (1967). 10.1093/genetics/55.3.513 PMC1211407 6068403

[14] Klevecz RR (1976). Quantized generation time in mammalian cells as an expression of the cellular clock. Proc. Natl. Acad. Sci..

[15] Robbins E, Scharff MD (1967). The absence of a detectable G1 phase in a cultured strain of Chinese hamster lung cell. J. Cell Biol..

[16] Das, M., Dempsey, E., Reeves, J., Stenmark, K. Selective expansion of fibroblast subpopulations from pulmonary artery adventitia in response to hypoxia. *Am. J. Physiol. Lung Cell. Mol. Physiol.* **282,** L976–L986 (2002). 10.1152/ajplung.00382.2001 11943662

[17] Cao Y, Kimura S, Itoi T, Honda K, Ohtake H, Omasa T (2012). Construction of BAC-based physical map and analysis of chromosome rearrangement in Chinese hamster ovary cell lines. Biotechnol. Bioeng..

[18] Arnold JN, Wormald MR, Sim RB, Rudd PM, Dwek RA (2007). The impact of glycosylation on the biological function and structure of human immunoglobulins. Annu. Rev. Immunol..

[19] Eon-Duval A, Broly H, Gleixner R (2012). Quality attributes of recombinant therapeutic proteins: An assessment of impact on safety and efficacy as part of a quality by design development approach. Biotechnol. Prog..

[20] van Berkel PH, Gerritsen J, Perdok G, Valbjørn J, Vink T, van de Winkel JG (2009). N-linked glycosylation is an important parameter for optimal selection of cell lines producing biopharmaceutical human IgG. Biotechnol. Prog..

[21] Yamano N, Kimura T, Watanabe-Kushima S, Shinohara T, Nakano T (2010). Metastable primordial germ cell-like state induced from mouse embryonic stem cells by Akt activation. Biochem. Biophys. Res. Commun..

[22] Shridhar S, Klanert G, Auer N, Hernandez-Lopez I, Kańduła MM, Hackl M (2017). Transcriptomic changes in CHO cells after adaptation to suspension growth in protein-free medium analysed by a species-specific microarray. J. Biotechnol..

[23] Hunter M, Yuan P, Vavilala D, Fox M (2019). Optimization of protein expression in mammalian cells. Curr. Protoc. Protein Sci..

[24] Xu, J., Tang, P., Yongky, A., Drew, B., Borys, M. C., Liu, S., *et al*., editors. Systematic development of temperature shift strategies for Chinese hamster ovary cells based on short duration cultures and kinetic modeling. *MAbs.* **11,** 191–204 (2019). 10.1080/19420862.2018.1525262 PMC6343780 30230966

[25] Yamano N, Omasa T (2018). EGCG improves recombinant protein productivity in Chinese hamster ovary cell cultures via cell proliferation control. Cytotechnology.

[26] Omasa T, Cao Y, Park JY, Takagi Y, Kimura S, Yano H (2009). Bacterial artificial chromosome library for genome-wide analysis of Chinese hamster ovary cells. Biotechnol. Bioeng..

[27] Yamano N, Takahashi M, Haghparast SMA, Onitsuka M, Kumamoto T, Frank J (2016). Increased recombinant protein production owing to expanded opportunities for vector integration in high chromosome number Chinese hamster ovary cells. J. Biosci. Bioeng..

[28] Onitsuka M, Omasa T (2015). Rapid evaluation of N-glycosylation status of antibodies with chemiluminescent lectin-binding assay. J. Biosci. Bioeng..

[29] Omasa T, Higashiyama K, Shioya S, Suga K (1992). Effects of lactate concentration on hybridoma culture in lactate-controlled fed-batch operation. Biotechnol. Bioeng..

[30] Kaneyoshi K, Yamano-Adachi N, Koga Y, Uchiyama K, Omasa T (2019). Analysis of the immunoglobulin G (IgG) secretion efficiency in recombinant Chinese hamster ovary (CHO) cells by using Citrine-fusion IgG. Cytotechnology.

