# Supplementary material for: Establishment of fast-growing serum-free immortalised cells from Chinese hamster lung tissues for biopharmaceutical production
Source: Sci Rep. 2020 Oct 19;10:17612. doi: 10.1038/s41598-020-74735-0 (PMC7572389; doi:10.1038/s41598-020-74735-0)

Establishment of fast-growing serum-free immortalised cells from Chinese hamster lung tissues for biopharmaceutical production

Noriko Yamano-Adachi[1, 2*], Rintaro Arishima[1], Sukwattananipaat Puriwat[1], and Takeshi Omasa[1, 2]

[1]Graduate School of Engineering, Osaka University, 2-1, Yamadaoka, Suita, Osaka 565-0871, Japan

[2]Manufacturing Technology Association of Biologics, 7-1-49, Minatojima-Minamimachi, Chuo-ku, Kobe, Hyogo 650-0047, Japan

Corresponding author:

Noriko Yamano-Adachi, Ph.D., Phone: +81-6-6879-4157, Fax: +81-6-6879-4157

Email: yamanori@bio.eng.osaka-u.ac.jp

Supplementary figure legends

Supplementary Fig. 1. Full-length gel images Fig. 2b
100 bp DNA Ladder (Takara Bio Inc., Shiga, Japan) was used for DNA size marker.

Supplementary Fig. 2. Full-length gel images of Fig. 3a
Gene Ladder Wide 1 (0.1-20 kbp) (NIPPON GENE CO., LTD., Tokyo, Japan) was used for DNA size marker.

Supplementary Fig. 1

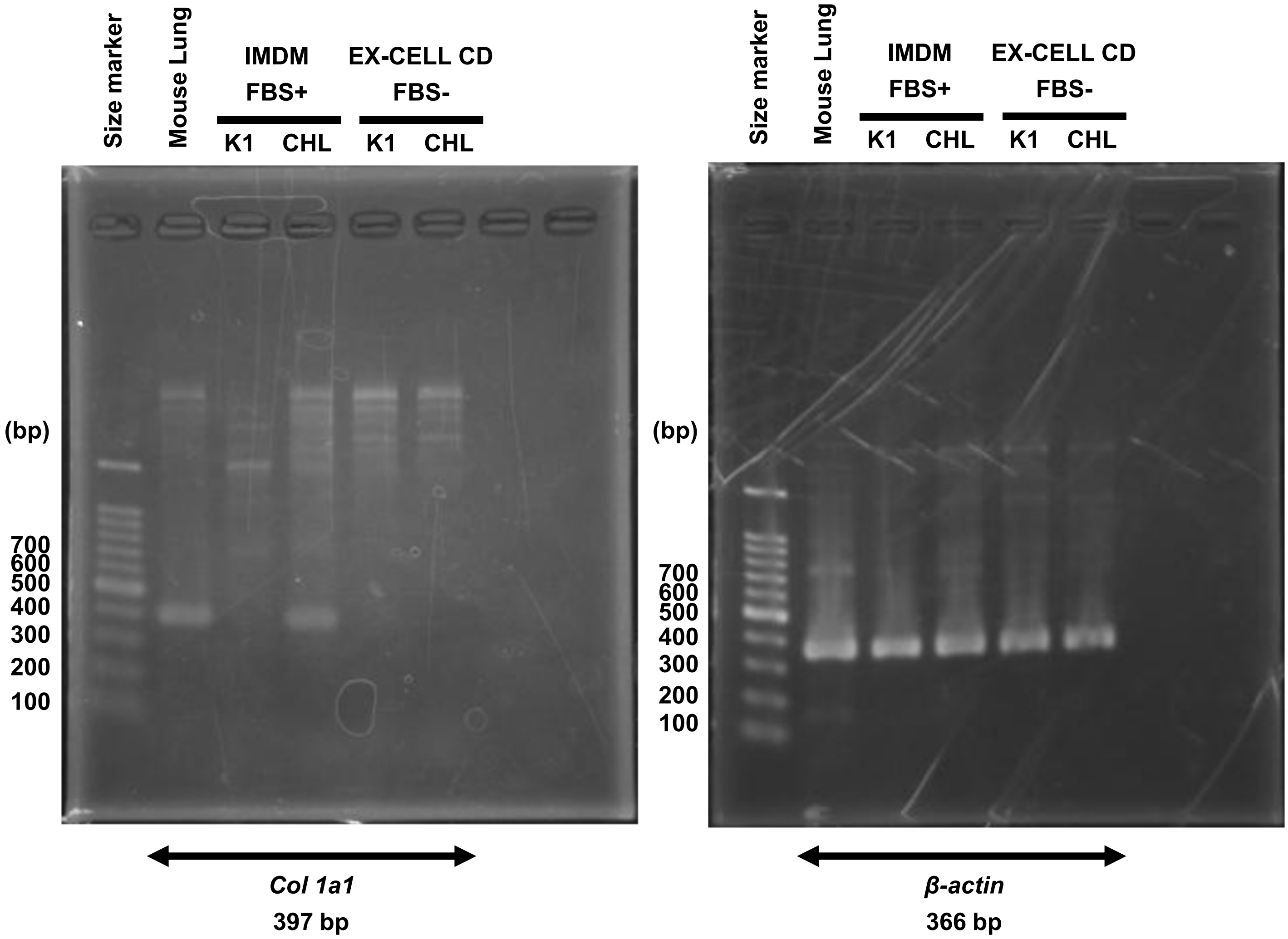

Supplementary Fig. 2

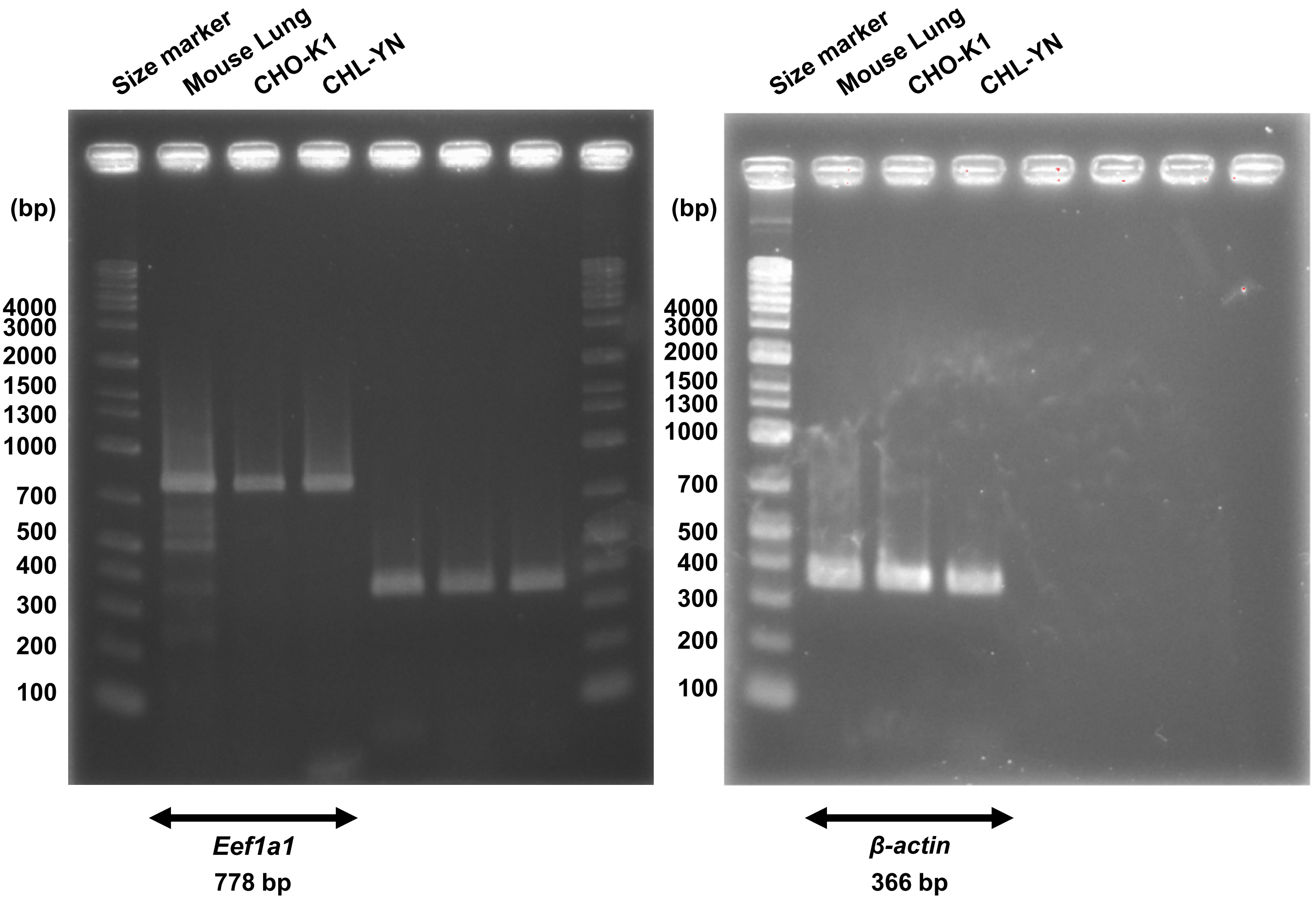

Supplement: Supplementary file 1 — Supplementary Information. [file 41598_2020_74735_MOESM1_ESM.pdf]
